# Mynodbcsv: Lightweight Zero-Config Database Solution for Handling Very Large CSV Files

**DOI:** 10.1371/journal.pone.0103319

**Published:** 2014-07-28

**Authors:** Stanisław Adaszewski

**Affiliations:** 1 Laboratoire de Recherche en Neuroimagerie, DNC, CHUV, Lausanne, Switzerland; 2 Blue Brain Project, EPFL, Lausanne, Switzerland; J. Craig Venter Institute, United States of America

## Abstract

Volumes of data used in science and industry are growing rapidly. When researchers face the challenge of analyzing them, their format is often the first obstacle. Lack of standardized ways of exploring different data layouts requires an effort each time to solve the problem from scratch. Possibility to access data in a rich, uniform manner, e.g. using Structured Query Language (SQL) would offer expressiveness and user-friendliness. Comma-separated values (CSV) are one of the most common data storage formats. Despite its simplicity, with growing file size handling it becomes non-trivial. Importing CSVs into existing databases is time-consuming and troublesome, or even impossible if its horizontal dimension reaches thousands of columns. Most databases are optimized for handling large number of rows rather than columns, therefore, performance for datasets with non-typical layouts is often unacceptable. Other challenges include schema creation, updates and repeated data imports. To address the above-mentioned problems, I present a system for accessing very large CSV-based datasets by means of SQL. It's characterized by: “no copy” approach – data stay mostly in the CSV files; “zero configuration” – no need to specify database schema; written in C++, with boost [1], SQLite [2] and Qt [3], doesn't require installation and has very small size; query rewriting, dynamic creation of indices for appropriate columns and static data retrieval directly from CSV files ensure efficient plan execution; effortless support for millions of columns; due to per-value typing, using mixed text/numbers data is easy; very simple network protocol provides efficient interface for MATLAB and reduces implementation time for other languages. The software is available as freeware along with educational videos on its website [4]. It doesn't need any prerequisites to run, as all of the libraries are included in the distribution package. I test it against existing database solutions using a battery of benchmarks and discuss the results.

## Introduction

When considering processing of big and wide data, emphasis is often put on custom solutions (e.g. scripts in MATLAB/R/Python, programs in C/C++, different NoSQL [5] solutions) that promise performance and customizability traditionally unavailable to normalized solutions like SQL-capable relational databases. However, it's worth realizing the benefits of using a standardized language for querying the data. Those include: shorter development time, maintainability, expressive and natural way of formulating queries, ease of sharing them with collaborators who need just to understand SQL to know the purpose of a query. Additionally, with scripting approaches to big data even reading the data source is frequently not easy because of inefficiency of high-level languages in running parsers. In light of these facts, it seems that reasons stopping potential users from choosing a database approach to handling their data are: inability of the latter to accommodate modern dataset sizes (big data) and layouts (wide data), necessity to install appropriate software and move data into the system, as well as designing an appropriate database schema beforehand. However, as solutions satisfying needs of efficient ad-hoc access to computationally demanding datasets using standard languages like SQL come to existence (NoDB [6], mynodbcsv), this situation becomes likely to change.

Problems described above have been previously studied in the field of database research. Among the better explored ones are those of auto-tuning – offline [7]–[16] and online [17], [18] and adaptive indexing [19]–[25]. Mynodbcsv satisfies both philosophies online, albeit it relies on a very simplistic, however effective strategy – it greedily indexes all columns used in dynamic (arithmetic/functional/conditional expressions, WHERE, ORDER BY, GROUP BY and JOIN clauses) parts of the queries. At risk of being suboptimal this design choice gives one significant benefit to the end-user – predictability. Each time a column is used for the first time in a dynamic way, it will be indexed.

Information extraction for the static part of the query is done using optimized CSV lookup algorithm, described in Table 1. The solution for integrating SQL semantics with unstructured text data retrieval as described in [26] is not required in case of mynodbcsv - since dynamic part of the query (therefore all the computationally demanding tasks of joining and filtering the data) is handled by SQLite, my software is limited to retrieving corresponding rows/columns from the static part using optimized linear scan.

**Table 1:** CSV Access Algorithm.

1	Load or map CSV file as-is into memory.
2	Perform initial parsing of the file, caching starting position of every 100^th^ column in each row, note: this doesn't parse the numbers etc. it only traverses the file to cache column positions.
3	Initialize cursor for each row to point to the first column.
4	If data query accesses column pointed by cursor of the respective row, parse it starting from the cursor and advance cursor by one column.
5	Else: look for the closest cached column position and find the destination column by dynamically parsing CSV, read the column and set cursor for that row to point to the next column.

In-situ processing as described in [27]–[33] is reinforced in mynodbcsv compared to previous accomplishments by its completely zero-config nature. Schemas are built automatically assuming that first rows of CSV files contain column names. New CSV files are introduced to the system by dragging and dropping them over the GUI or using a classical file selection dialog. Their names are converted to table names. SQL queries are instantly possible for all new data.

## Materials and Methods

A good example of almost entirely CSV-based dataset is the tabular data from Alzheimer's Disease Neuroimaging Initiative (ADNI) [34]. A subset of it containing roughly 130 CSV files with clinical data about subjects was frequently used in neuroscience studies in recent years. In the first test, I tried to import all of the data into an SQLite database. This proved to be efficient for querying but the import process itself was slow. The necessity to prepare a schema beforehand and re-import each file whenever certain kinds of change of the original data were made (e.g. inserting new records, performing global text processing) was also a hassle. Another type of data that ADNI provides is structural magnetic resonance imaging (sMRI) data of the brain. After pre-processing those data, there were about 400000 features for each scan, corresponding to voxels of gray matter. With this amount of columns no database solution at my disposal could handle it. At the same time, I realized that the set of interesting queries requiring all of the above data combined was limited. It consisted mostly of simple filtering, grouping and ordering using subject's diagnosis, age or gender as criteria. This notion called for a more efficient way of accessing the data, which didn't require loading all of it into the database but rather reduced the imported parts to absolute minimum, i.e. just the columns used in SQL's WHERE/GROUP BY/ORDER BY clauses and in the dynamic expressions in the SELECT clause, while obtaining the rest of the data directly from the original CSV files.

**Figure 1:**
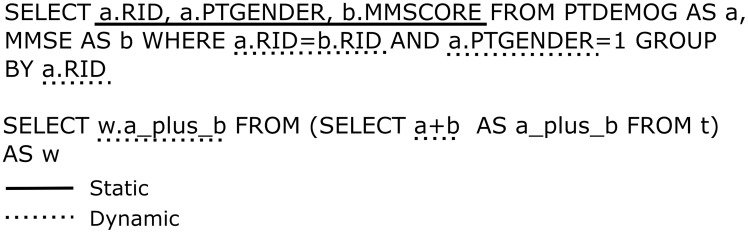
Examples of static and dynamic column references. Dynamic references are kept in the reformatted query, whereas for static ones the identifier column of the corresponding table is added and subsequently they are fetched directly from the original CSV file.

**Figure 2:**
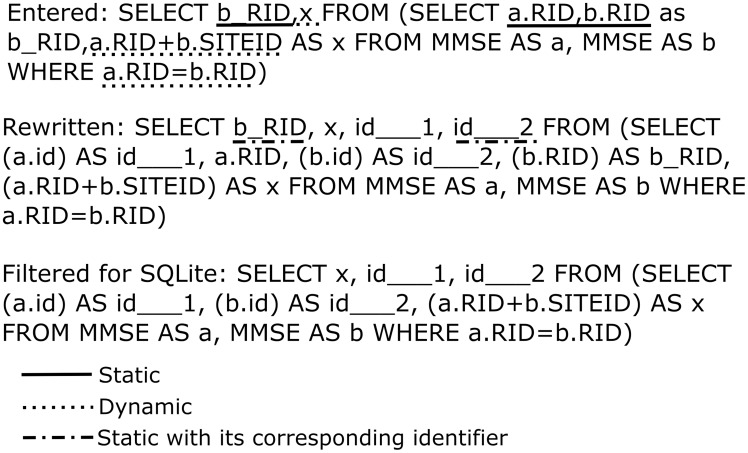
Query rewriting pipeline. In the first step, wildcard expressions are expanded and necessary identifier columns added. In the second step, static values are filtered out giving the query that is actually executed by SQLite.

**Figure 3:**
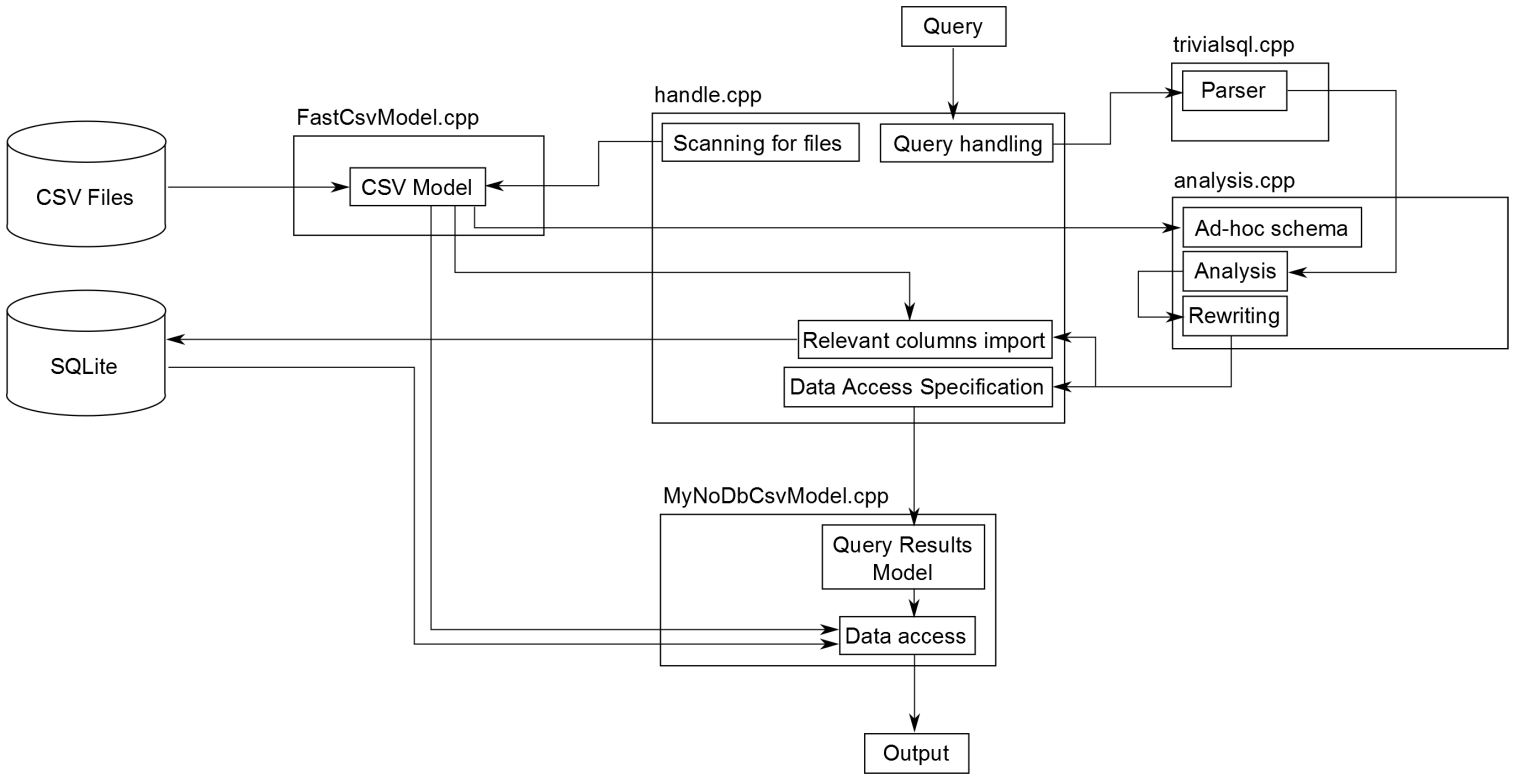
Block diagram of mynodbcsv architecture.

Achieving the above in a completely robust manner (e.g. supporting nested SELECT queries in the FROM clause, column aliases, JOINs) excluded any simple text processing and required writing a proper SQL parser. The idea was to restructure [Figures 1,2] the original query in such a way that only dynamic parts were retained and row identifiers added for the static parts which later could be used to fetch data from the original CSV files. For implementing the parser I used Boost Spirit parsing library and defined the syntax corresponding to SELECT syntax in SQLite. The parser takes a string containing an SQL query as input and produces the Abstract Syntax Tree (AST), which already specifies (to limited extent) which parts of the query are dynamic. Further analysis step is necessary to determine if expressions, which syntactically seem to be static are in fact dynamic because they come from nested dynamic SELECT queries. The analysis module detects these cases and for each dynamic identifier makes sure that “id” column of each of the tables used to produce that expression is included once in the list of SELECT values. These added identifiers are named by convention “id___N” where N is an increasing integer number for each new generated identifier. All “id___N” values are propagated across nested queries regardless whether they are used in the final output. The tables and columns are imported on-demand only for the dynamic parts of the query. This is the key to obtaining good performance. Temporary in-memory tables can be used to be even faster. After retrieving identifiers from the reformatted query results, original AST is used to fill in the missing static parts by accessing CSV files. Large CSV files support is achieved by keeping them mostly in memory (since file mapping is used to this end, the percentage of file loaded into physical memory depends on the amount of memory available and the file usage pattern) with minimum overhead for caching some of the column positions. Afterwards, columns are accessed by parsing the file on the fly, using cached positions to amortize search time for each particular column. This proved to be more efficient both performance- and memory-wise than keeping parsed data in arrays of strings or variant types. Finally, results are either printed out as CSV file, presented by means of a dedicated graphical user interface (GUI) implemented using the Qt library and a special data model (derived from QAbstractItemModel class) or sent in CSV format over a network socket. This approach is more robust than trying to wrap the algorithm in an existing database interface (either native SQLite or a generic one like ODBC) and provides the necessary performance level to do online analysis of all the results. Wrapping it in an existing API would have had an added benefit of offering a drop-in replacement functionality for existing applications but I chose to prioritize implementation time, robustness and speed. My solution doesn't require schema specification. All CSV files found in the current working directory are automatically seen as tables in the database with all the necessary columns imported on-demand. Further files can be added using a drag-and-drop interface or from the menu. For an overview of the architecture, see [Figure 3].

I tested mynodbcsv against three established database management systems (DBMS), in order of decreasing similarity to mynodbcsv – HSQLDB [35] (support for unlimited columns and CSV data storage), H2 [36] (unlimited columns, requires data import) and PostgreSQL [37] (limited columns, requires import). When possible I created indices for columns used in WHERE clauses. HSQLDB failed to create such indices for CSV-backed tables within 10 minutes, so I proceeded without them.

In order to evaluate basic function of my implementation I performed a set of queries on the abovementioned ADNI dataset including genetic data (about 30000 single nucleotide polymorphisms (SNPs)) and imaging dataset reduced using custom atlas from 400000 to 6800 features – an approach I used before to store the data in a PostgreSQL database. Even after such reduction it was impossible to store the data in a single table in PostgreSQL because of the built-in limit of (depending on the type) 250-1600 columns per table. Therefore, I decided to store only columns that were going to be used in expressions and put the remaining data without modifications in one additional column named “rest”.

Furthermore, to demonstrate its performance on big data, I used Allen Brain Atlas single subject gene expression data (900 MB CSV file, 60000 rows, 1000 columns), as well as an artificially generated file with 1000 rows and 400000 columns mimicking the situation with original imaging data. The latter scenario exceeded beyond what was possible with existing database solutions. I didn't perform it using other databases because of the vast performance gap between them and mynodbcsv, which would require too much time to complete.

All of the above tests were executed under Windows 7 operating system running on a PC with 16GB of memory and Intel Core i5-2400 processor running at the frequency of 3.10 GHz. Code was compiled with optimizations using GCC C++ Compiler version 4.6.2.

Furthermore, in order to place mynodbcsv in relation to NoDB, I performed a test similar to the first microbenchmark in [6] on a Macbook Air 13” 2013 model with 8 GB of RAM, Intel i7 CPU at 1.8 GHz and an SSD hard drive. Benchmark data consisted of 7.5 million rows with 150 columns containing integers in the range [0, 10^9^). 10 SELECT queries without WHERE clause were executed with 10 random columns each. Next, 10 SELECT queries on 10 random columns with WHERE clause for one random column, were executed.

The abovementioned microbenchmark is representative of “narrow” data performance. Since this is the case, I decided to include also traditional (MySQL [38]) and innovative (wormtable [39], Teiid [40]) database systems, which were created for handling datasets with vertical extent much bigger than the horizontal one. This comparison further illustrates mynodbcsv's position in the database landscape.

In order to save storage space (in excess of 1 gigabyte) and bandwidth, mock-up versions of data files necessary to reproduce all of the above tests can be generated by scripts in File S1. Copies of original data (where applicable) are available from the ADNI [34] and Allen Brain Atlas [41] websites.

## Results

In the “wide” data benchmarks, mynodbcsv was the only truly satisfactory solution feature-wise, most of the time outperforming competitors also performance-wise [Tables 2,3,4].

**Table 2:** Performance comparison of mynodbcsv and HSQLDB/H2/PostgreSQL.

Query/Database	mynodbcsv	PostgreSQL*	HSQLDB**	H2
SELECT * FROM ADNI_Clin_6800_geno	347.9±8.79	3978.1±30.27	43579.0±1432.11	35412.0±1611.24
SELECT * FROM ADNI_Clin_6800_geno WHERE Diagnosis = ” AD “	436.1±10.88	702.7±13.0618	30513.0±5064.80	7543.2±167.83
SELECT * FROM ADNI_Clin_6800_geno WHERE Diagnosis = " AD " OR PTGENDER = 1 OR RID%2 = = 0	689.2±12.02	3046.6±33.58	38849.5±889.83	20650.1±946.79
SELECT * FROM ADNI_Clin_6800_geno AS a INNER JOIN PTDEMOG AS b USING(RID) WHERE a.Diagnosis = " AD " OR a.PTGENDER = 1 OR a.RID%2 = = 0	771.9±6.22	4830.6±48.37	262336.7±9080.58	11206.3±527.54

All execution times in [ms].

*For PostgreSQL only columns necessary for testing the WHERE/JOIN/etc. conditions were created in respective tables, the remaining columns where preserved in CSV format in one column called “rest”. Time required for parsing of the CSV column is not included in the measurements as it would be negligibly small compared to the query execution time. ** For HSQLDB the actual CSV support was used.

**Table 3:** Performance comparison of mynodbcsv and HSQLDB/H2/PostgreSQL using single subject Allen Brain Atlas data.

Query/Database	mynodbcsv	PostgreSQL	HSQLDB	H2
SELECT * FROM MicroarrayExpression_fixed AS a INNER JOIN Probes AS b ON(a.c1 = b.probe_id)	310.9±14.77	72158.9±1312.98	Didn't finish	75680.8±20192.18

All execution times in [ms].

**Table 4:** mynodbcsv performance on a table with 400000 columns and 1000 rows (dummy); dummy2 has 10000 columns and 1000 rows.

Query	Mynodbcsv Execution time [ms]
SELECT * FROM dummy	6297.8±47.04
SELECT * FROM dummy AS a INNER JOIN dummy2 AS b USING(c0)	7391.2±123.261

It could be seen that performance of PostgreSQL was severely reduced when serving big TEXT-type column. In order to determine the root cause of this inefficiency I've run PostgreSQL with its data folder placed on a RAM disk created using ImDisk Virtual Disk Driver to remove any unfair advantage of mynodbcsv keeping data in-memory all the time. The difference in timings for PostgreSQL was negligible, therefore, I didn't report the second timings. I suppose that this poor performance might be a result of sockets-based API of PostgreSQL, which has to perform reformatting necessary for the protocol and push large volumes of data through a loopback network connection. Mynodbcsv on the other hand has direct access to the data. This model is better suited for local access and doesn't constitute a security issue if shared memory with appropriate protection flags is used to exchange data between different processes.

Mynodbcsv had a tendency for query time increase as function of number of columns. This performance hit was caused by the query rewriting mechanism which tracks all of the columns coming from tables in FROM and JOIN clauses throughout the query. It could be further optimized to either remove tracking of columns not specified explicitly in the SELECT clause (e.g. when “*” is used) or to decrease the computational complexity of column tracking. Removing this overhead would offer an order of magnitude increase in efficiency in some situations. Noteworthy, for image file formats where numbers of columns can reach millions, a very efficient array-addressing extension was introduced to the syntax, allowing column access using offset specification instead of name lookup. This practically eliminated the above problem.

Overall, mynodbcsv was the only solution to offer this level of performance and not suffer from any limitations.

In the first microbenchmark results [Tables 5,6], it took about 120 s for mynodbcsv to perform initial file scan and then 34 s for the 1^st^ query. The second query took 8.4 s. Successively the query time stabilized at about 4.5 s. Probably NoDB would be significantly better than mynodbcsv in the other benchmarks used to evaluate it against existing DBMSes in [6] because queries containing projections/aggregations on all of the attributes would force mynodbcsv to build full SQLite database with all of the columns. Overcoming this is impossible in the current “intermediate” framework of mynodbcsv, however in my experience this is 1) a rare scenario, 2) can be easily circumvented by streaming query results to a custom application instead of projecting/aggregating on the DB side.

**Table 5:** Query time comparison in microbenchmark similar to the first one from [6], without WHERE condition.

Database/Run	Initial Load	1	2	3	4	5
Mynodbcsv	∼120	34.165	8.441	7.949	7.780	5.373
MySQL	∼285	178.706	185.047	175.109	185.564	189.746
Wormtable	∼583	1357.733	1343.813	1342.653	1456.482	1418.348
Teiid	∼0	602.970	625.252	665.062	637.091	657.034

Database/Run	6	7	8	9	10	Mean±SD*
Mynodbcsv	5.364	5.219	5.321	4.583	4.408	8.860±8.549
MySQL	185.712	180.901	183.235	193.692	185.334	184.305±5.015
Wormtable	1614.710	1469.741	1412.251	1348.159	1347.477	1411.137±81.902
Teiid	692.183	644.607	625.928	616.335	642.654	640.912±24.529

All times in [s].

*Without Initial Load time.

**Table 6:** Query time comparison in microbenchmark based on first one from [6], with WHERE condition.

Database/Run	1	2	3	4	5	
Mynodbcsv	235.406	215.238	221.219	223.249	227.089	
MySQL	100.214	106.100	99.777	98.810	99.280	
Wormtable	1378.221	1329.387	1372.538	1419.567	1644.511	
Teiid	571.458	585.293	620.953	622.774	632.024	

Database/Run	6	7	8	9	10	Mean±SD
Mynodbcsv	237.116	238.105	248.184	210.987	219.704	227.630±11.120
MySQL	102.006	104.829	113.525	103.399	107.366	103.530±4.366
Wormtable	1488.179	1641.428	1872.519	1510.913	1646.998	1530.426±160.910
Teiid	615.900	775.444	835.995	847.338	713.459	682.064±98.207

All times in [s].

In the second test, mynodbcsv suffered a bit from on-the-fly parsing of CSVs which was tuned for parsing wide rather than long data. It didn't approach the performance of NoDB, falling behind by a factor of approximately 4 times. Perhaps different hardware configurations also affected the results in favor of NoDB.

Compared to wormtable and Teiid, mynodbcsv was again outperforming the other two by 1–2 orders of magnitude in both microbenchmark variants. When it comes to MySQL, mynodbcsv was an order of magnitude faster in the first variant (without WHERE clause) and about two times slower when the WHERE clause was present. This is yet again attributable to the way the benchmark was constructed, forcing mynodbcsv to add a new column to its SQLite store in each run.

## Discussion

The idea of using textual format for database storage isn't new and has been implemented completely or partially in existing solutions already. Mynodbcsv, however, is using an intermediate approach between building a new database engine and importing data to existing one. Doing so using query rewriting provides an efficient, robust and lightweight solution for many typical use-cases. It manages to reduce standard database involvement to the minimum and accesses bulk of the data directly in CSV files.

As can be seen in comparison to NoDB, it shares many of the same on-the-fly parsing mechanisms, however as it is less coupled with the query, it follows a more greedy approach when deciding what to parse (i.e. entire columns of data).

Since mynodbcsv uses an existing database engine without any modifications it can be configured with different backends. SQLite has been chosen as a particularly lightweight and standalone solution, however any database with appropriate Qt connector could be a drop-in replacement. For example, a binding to PGSQL (PostgreSQL) is a work in progress. It will be useful in certain situations because PGSQL supports FULL OUTER JOIN semantics, while SQLite doesn't.

Support for CSV as data storage format is also not the only option. The engine itself is completely unaware of using them, as they are represented transparently with a QAbstractItemModel interface. Mynodbcsv is easily extensible in terms of supported data formats, as long as similar representation is possible for them. Such a mapping for Nifti [42] files is a work in progress and future possibilities include also Hierarchical Data Format (HDF5), Extensible Markup Language (XML), MATLAB file format and others.

HSQLDB was the only freely available competitor offering support for unlimited number of columns and CSV-based tables. However, in the benchmarks it fell a long way behind both mynodbcsv and PostgreSQL in terms of performance. Having support for “real” columns and being able to execute functions on them as part of the query gives HSQLDB certain advantage, but in my experience with such wide tables, the typical use case is not to analyze them entirely within a database query. Usually it's rather a matter of data integrity. Robustness benefits from keeping everything in a single table, without resorting to cross filesystem linking. It's also convenient to retrieve selected parts of data by name. I tried to improve HSQLDB performance by creating indices on the columns used in WHERE clauses of the queries. This operation was taking an indefinite amount of time when text table sources were attached, so following the software documentation I detached them, created the indices and reattached the sources. However after this operation, queries involving JOIN clause started failing with “unsupported internal operation RowStoreAVL” error message. Therefore, results for HSQLDB are given without using indices.

H2 was significantly faster than HSQLDB with its support for unlimited columns when data were imported into the database, yet it was still far from the performance offered both by PostgreSQL and mynodbcsv. Also its limitation of only one process accessing the database at any given time was problematic already during testing and most probably would escalate in production environment.

Although mynodbcsv was slower than NoDB for some queries, it's noteworthy that at the same time it used only 2 * 7500000 * 4 = 60 MB of additional lookup space, with everything else effectively staying in the CSV files.

Mynodbcsv managed as well to outperform interesting and innovative solutions like wormtable and Teiid in the microbenchmark tests, falling behind MySQL slightly in the second benchmark variant. This deficiency could be mitigated by improving performance of SQLite imports, for example by importing columns into separate tables and joining them using views as opposed to re-creating a new table with all the necessary columns each time as it is done now. More radical solutions involve embedding mynodbcsv's CSV support directly in SQLite or writing a custom query execution engine from scratch.

From the perspective of end-user, mynodbcsv is already a versatile tool - facilitating easy handling of big data stored in CSV files. Despite the above examples being biased towards neuroscientific research, it's a completely generic solution with applications that are much broader and in fact valid for any type of tabular or “convertible to tabular” data. Potential uses - scientific, industrial and personal include astronomy, physics, economy, education, public health and more.

One could consider adding SQL completion solutions such as SQLSUGG [43] to the GUI in order to offer a helping hand to the users with less SQL expertise. On the backend side, support for horizontal aggregations [44] seems like a great addition to the big data nature of mynodbcsv.

Network access to mynodbcsv is another potential area for improvement. Issues involved include formatting of the results, which at the moment is plain CSV but could be optimized using binary encoding and/or compression. Combining these two approaches would improve performance on the client side because of reduced network bandwidth and CSV processing overhead. The problem of database locking while importing columns is currently solved using a global mutex, which allows one client to block the others when running a query that requires importing of too much data. This could be solved either by switching from SQLite backend to one that allows simultaneous writes to a database or by creating a dedicated SQLite database for each connected client. For many scenarios the latter solution seems like a fast and reliable option.

## Conclusions

Processing very big and wide data is now commonplace in many professional environments. Ability to access it efficiently without building the usual database infrastructure is the holy grail of in-situ approach. Mynodbcsv offers the “best of both worlds” alternative for everybody who would like to benefit from in-situ SQL data processing without the hassle of setting up heavier and more elaborate systems. It's also to the best of my knowledge the only truly zero-config solution, which takes as little as drag and drop to attach data. Last but not least thanks to the portability of libraries used, it works out of the box on Windows, Linux and Mac OS X platforms. There are still many areas for improvement (tighter coupling of query analysis and data indexing/caching, better network performance, multi-threading), however current imperfections are counterbalanced by interesting properties of the system – speed, robustness, small footprint and predictability. I keep working on it so that one day it may join the family of next generation software solutions for data mining.

## Supporting Information

File S1 **Set of Python scripts to generate data for benchmarks: equivalents of ADNI_Clin_6800_geno.csv, PTDEMOG.csv, MicroarrayExpression_fixed.csv and Probes.csv files, the dummy.csv, dummy2.csv and the microbenchmark CSV files.**
(ZIP)
